# *Mycobacterium tuberculosis* peptide-specific T cells in pulmonary granulomas display broad effector functions

**DOI:** 10.1016/j.isci.2025.114034

**Published:** 2025-11-12

**Authors:** Christine E. Nelson, Keith D. Kauffman, Kevin C. Osum, Shunsuke Sakai, Jay Buchanan, Jean M. Chanchu, Melanie Cohen, Julie Laux, Iyadh Dougi, Katherine M. Barrows, Ifeanyichukwu U. Anidi, Cecilia S. Lindestam Arlehamn, Alessandro Sette, April Walker, Amirhossein Shamsaddini, Justin Lack, Joel D. Ernst, Carl G. Feng, Laura E. Via, Kevin P. Fennelly, Shamus R. Carr, Daniel L. Barber

**Affiliations:** 1 T Lymphocyte Biology Section, Laboratory of Parasitic Diseases, National Institute of Allergy and Infectious Diseases, National Institutes of Health, Bethesda, MD, USA; 2 Division of Pulmonary and Critical Care Medicine, Department of Medicine, University of Virginia, Charlottesville, VA 22903, USA; 3 Cytometry Section, Research Technologies Branch, National Institute of Allergy and Infectious Diseases, National Institutes of Health, Bethesda, MD, USA; 4 Surgical Oncology Program, National Cancer Institute, National Institutes of Health, Bethesda, MD, USA; 5 Critical Care Medicine and Pulmonary Branch, National Heart, Lung and Blood Institute, National Institutes of Health, Bethesda, MD, USA; 6 Center for Infectious Disease and Vaccine Research, La Jolla Institute for Immunology, La Jolla, CA, USA; 7 Center for Vaccine Research, Department of Infectious Disease Immunology, Statens Serum Institute, Copenhagen, Denmark; 8 Department of Medicine, University of California, San Diego, San Diego, CA, USA; 9 Tuberculosis Imaging Program, Division of Intramural Research, National Institute of Allergy and Infectious Disease, National Institutes of Health, Bethesda, MD, USA; 10 Collaborative Bioinformatics Resource, Research Technologies Branch, National Institute of Allergy and Infectious Diseases, National Institutes of Health, Bethesda, MD, USA; 11 Division of Experimental Medicine, Department of Medicine, University of California, San Francisco, San Francisco, CA, USA; 12 Immunology and Host Defense Group, Faculty of Medicine and Health, The University of Sydney, Sydney, NSW, Australia; 13 Tuberculosis Research Program, Centenary Institute, The University of Sydney, Sydney, NSW, Australia; 14 Tuberculosis Research Section, Laboratory of Clinical Immunology and Microbiology, Division of Intramural Research, National Institute of Allergy and Infectious Disease, National Institutes of Health, Bethesda, MD, USA; 15 Thoracic Surgery Branch, National Cancer Institute, National Institutes of Health, Bethesda, MD, USA

**Keywords:** immunology, microbiology, transcriptomics

## Abstract

*Mycobacterium tuberculosis* (Mtb) peptide-specific T cell responses in granulomas are essential for host protection. Here we profiled macaque and human granuloma Mtb-specific T cells with single cell RNA sequencing after stimulation with peptide pools. Mtb peptide-specific T cells are primarily Th1^∗^ cells and express pro-inflammatory cytokines, mutiple chemokines, TNF superfamily molecules, granzyme B and perforin, SLAM family molecules, semaphorins, growth factors, proteases, and collagen. Peptide recognition by Mtb-specific T cells also drives responses in bystander and unconventional T cells including expression of *FLT3LG*, *LTA*, and *XCL1*. In granulomas from a patient that underwent a pneumonectomy to remove tuberculosis-destroyed lung, we identify a population of peptide-specific T cells similar to macaque peptide-stimulated T cells. Thus, Mtb peptide-specific T cells may mediate protection against Mtb infection through the combined effects of many functions as well as the induction of bystander responses by neighboring T cells.

## Highlights

• NHP and human granuloma Mtb-specific T cells were identified by scRNAseq after stimulation
• NHP and human Mtb-specific T cells are mostly Th1∗ cells and functionally very similar
• They express cytokines, chemokines, TNF and SLAM family molecules, semaphorins, and collagen
• Peptide recognition by Mtb-specific T cells drives effector responses in bystander T cells

## Graphical abstract

**Figure:**
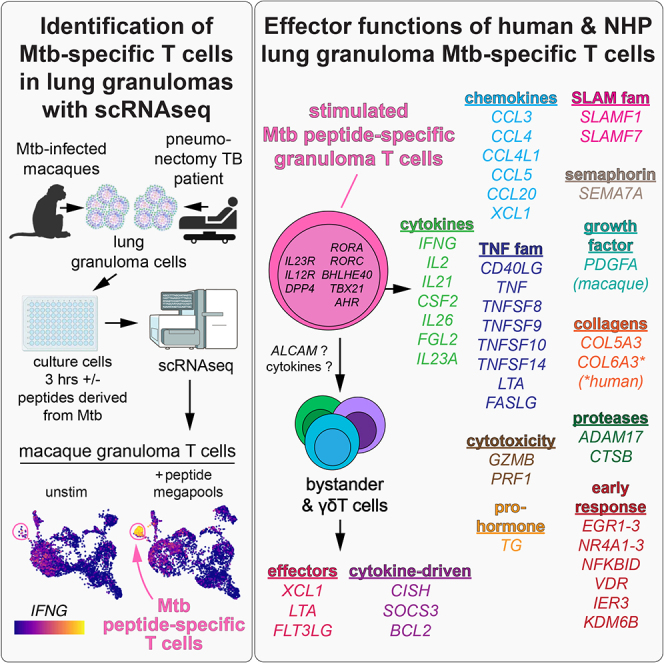

## Introduction

*Mycobacterium tuberculosis* (Mtb) infection is the leading cause of death due to infectious disease,^1^ and a highly protective vaccine against Mtb is needed to reduce the burden of disease. T cell responses are essential for control of Mtb infection and will likely be a central part of an effective vaccine. Much of our understanding of peptide-specific T cell differentiation, polarization, effector functions, regulation, and roles in protection is derived from murine model studies using common inbred strains like C57Bl/6 and BALB/c mice. These mouse strains generate strongly polarized Th1 responses against Mtb infection which does not always reflect the class of T cell response generated in humans.^2^^,^^3^^,^^4^ In humans and macaques, Mtb infection elicits peptide-specific T cells characterized as either Th1 cells or as cells with a mixture of Th1 and Th17 characteristics (referred to as Th1^∗^ cells). Th1^∗^ cells are associated with protection from TB.^5^^,^^6^^,^^7^^,^^8^^,^^9^^,^^10^^,^^11^ Indeed, individuals with deficiencies in *TBX21*, *IL12RB1*, *RORC*, *IL23R*, or those with anti-IL-23 neutralizing autoantibodies are susceptible to Mtb infection.^12^^,^^13^^,^^14^^,^^15^^,^^16^^,^^17^ Thus, while the diversity of Mtb peptide-specific T cell effector polarization states in humans and macaques is incompletely understood, it largely comprises various populations of IFNγ-producing cells.

T cell-dependent protection against Mtb has long been attributed to their production of IFNγ. There is data showing IFNγ-independent protection against Mtb infection by T cells.^18^^,^^19^^,^^20^^,^^21^ Tumor necrosis factor (TNF) and granulocyte-macrophage colony-stimulating factor (GM-CSF) may contribute to T cell-mediated protection, but no effector molecule other than IFNγ has been shown to be essential for T cell-mediated control of Mtb infection.^22^^,^^23^^,^^24^^,^^25^ This may be in part due to the lack of information on the function and specificity of granuloma T cells. Bulk RNAseq analysis of lung and blood T cells from Mtb-infected macaques has identified genes preferentially expressed in granuloma vs. circulating T cells.^26^ Single cell RNA sequencing (scRNAseq) analysis has identified several distinct T cell subsets at sites of Mtb infection.^27^^,^^28^^,^^29^^,^^30^ Yet, it is not clear from these data which populations of T cells are Mtb-specific and which are bystanders, as several studies have found that only ∼1%–20% of T cells in granulomas are Mtb peptide-specific.^31^^,^^32^^,^^33^ Thus, the mechanisms T cells use to suppress Mtb growth at sites of infection are poorly understood, largely due to the lack of functional data on Mtb peptide-specific T cells in granulomas.

Here we characterize the functional profiles of Mtb-specific granuloma T cells stimulated with cognate antigens. *Ex vivo* stimulation with a pool of several hundred Mtb-derived, T cell-antigenic peptides triggers major transcriptional changes in the Ag-specific T cells, making them easily identifiable in scRNAseq data. Upon T cell receptor (TCR) stimulation, we find that macaque Mtb peptide-specific T cells rapidly express many potentially disease-relevant genes, encompassing a broad array of effector functions. Mtb peptide-specific T cells also drive robust responses in non-specific bystander and unconventional T cells, indicating that conventional peptide-specific T cells may also protect against Mtb infection by promoting effector functions of unconventional T cells. Lastly, we examine the function of granuloma T cells isolated from a surgically resected lung from a TB patient and find a population of T cells that closely matches macaque Mtb peptide-specific T cells. These data give insight into the properties of Mtb peptide-specific T cells and provide novel candidate molecules that may play a role in T cell-mediated protection and pathology in TB.

## Results

### Mtb peptide-specific T cells in granulomas co-express many effector molecules

**Figure 1:**
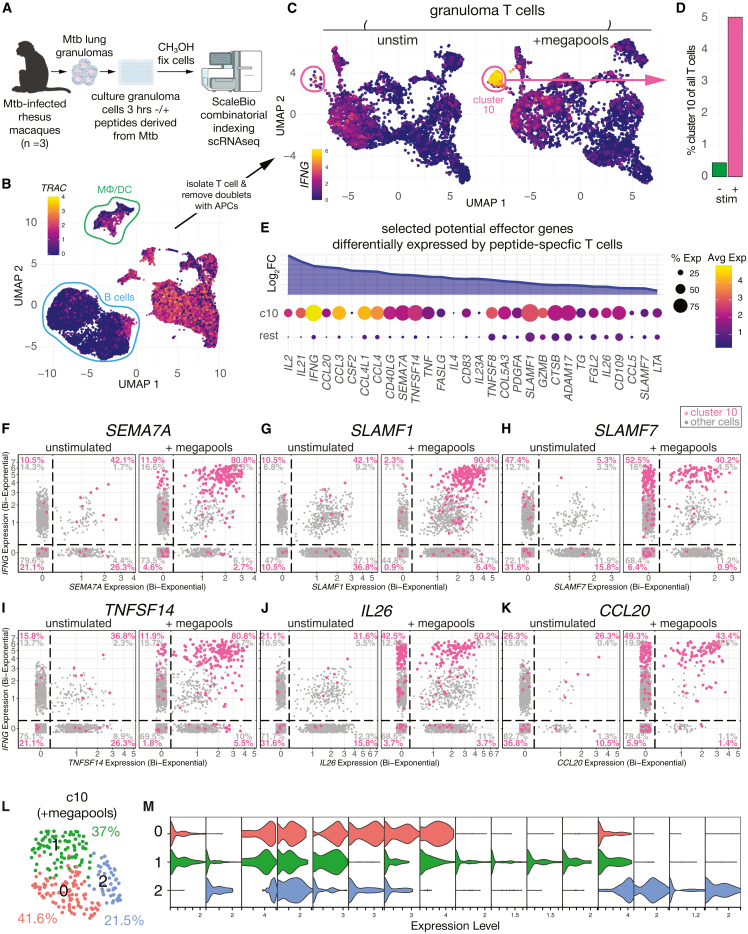
Mtb peptide-specific T cells in granulomas co-express many effector molecules after stimulation by cognate antigen (A) Granuloma cells from rhesus macaques were stimulated with Mtb peptide megapools for 3 h and combinatorial indexing scRNAseq analysis was performed. (B) *TRAC* expression by granuloma T cells. (C) T cells were identified as *TRAC*+ or *CD3E*+ and negative for *CD19*, *MS4A*, *CD68*, *IDO1*, *FLT3*, *FCRL5*, *CD20*, and *CIITA*, to remove a significant number of presumptive T cell: APC conjugates we observed with this technique. Plot shows *IFNG* expression on T cells cultured without or with the Mtb MTB300 and CD8 Mtb peptide megapools. Cluster 10 is highlighted as a distinct population of *IFNG*^high^ cells that appears after peptide stimulation. (D) Percentage of cluster 10 cells among T cells without or with peptide megapool stimulation. (E) Plot shows selected genes, primarily with putative effector or regulatory function, that were differentially expressed higher by cluster 10 cells compared to all other T cells. All genes shown were adjP <0.05. (F–K) Bivariate plots of *SEMA7A* (F), *SLAMF1* (G), *SLAMF7* (H), *TNFSF14* (I), *IL26* (J), and *CCL20* (K) each versus *IFNG*. Cluster 10 cells are highlighted in pink and all other T cells are plotted in gray. To facilitate visualization of gene co-expression and cells with expression levels of 0, the axes have been bi-exponentially transformed and jitter added. (L) Cluster 10 cells were reclustered, yielding three subsets. Numbers represent the percentage of each subcluster among all cluster 10 cells. (M) Violin plot of selected genes separated by the three subsets of cluster 10 cells.

We sought to characterize the transcriptional response of Mtb-specific T cells to TCR triggering. Cryopreserved rhesus macaque granuloma cells were thawed, rested overnight, and live cells identified by fluorescence-activated cell sorting (FACS) were purified. Cells were cultured with or without MHC class I- and II-binding Mtb peptide megapools^34^ for three hours and then fixed in a methanol-containing solution from Scale Biosciences (Figure 1A). A scRNAseq library pool was generated and sequenced. In all, 24,793 rhesus macaque granuloma cells were obtained. Colony forming units (CFU) from the granulomas used for the scRNAseq were not measured. However, other granulomas from the same animals were plated for determination of bacterial loads, and the means±SDs were as follows: DI4D, 338 ± 183 CFU, DI5F, 608 ± 205 CFU, and DI6N, 293 ± 173 CFU. Most cells recovered were T cells and B cells, although smaller populations of macrophages and dendritic cells were also present (Figure 1B). After stimulation, Mtb peptide-specific T cells were easily identifiable as a drastically increased population of cells (cluster 10) expressing high levels of *IFNG* (Figure 1C). Cluster 10 increased ∼10-fold in frequency after stimulation (Figure 1D), comprising ∼5% of total T cells. Thus, similar to how antigen-specific T cells are typically detected by flow cytometry with intracellular cytokine staining for IFNγ after peptide-stimulation, we can identify peptide-responsive T cells by scRNAseq analysis after a brief *in vitro* stimulation. This allows us to characterize the transcriptome-wide response of Mtb-specific T cells to cognate peptide recognition.

Compared to other T cells, the peptide-specific T cells (cluster 10) expressed elevated levels of several potential effector genes (Figure 1E). These included cytokines: *IL2*, *IL21*, *IL26*, *IFNG*, *CSF2*, *PDGFA*, *IL23A*, and *IL4*; TNFSF family genes: *TNF*, *CD40LG*, *TNFSF14* (LIGHT), *TNFSF8* (CD153/CD30L), and *LTA*; chemokines: *CCL20*, *CCL3*, *CCL4*, *CCL4L1*, and *CCL5*; proteases: *CTSB* and *ADAM17*; and SLAMF family molecules: *SLAMF1* and *SLAMF7*. The Ag-specific cells also expressed transcripts for the semaphorin *SEMA7A* and the prohormone thyroglobulin (*TG*). These genes were co-expressed with *IFNG*, indicating that IFNγ-producing Mtb-specific T cells display a very broad set of immunological functions (Figures 1F–1K). The Mtb peptide-specific T cells could be subclustered into three subsets (Figure 1L). The most abundant cluster, cluster 0, mostly comprised *CD4*^+^ cells that expressed high levels of *IFNG*, *TNF*, *IL2*, *CCL20*, and *TNFSF8* (CD153/CD30L). Cluster 2 was predominantly *CD8A*^+^ cells that expressed *GZMB*, *KLRD1*, and *CCL5*. Cluster 1 was a mix of CD4 and CD8 T cells that were characterized by lower expression of *IFNG*, *TNF*, and *CD40LG*, but higher expression of *TNFRSF8* (CD30), *KLRB1* (CD161), and *TNFSF10* (TRAIL). Thus, Mtb-specific T cells are comprised of several functionally distinct populations.

**Figure 2:**
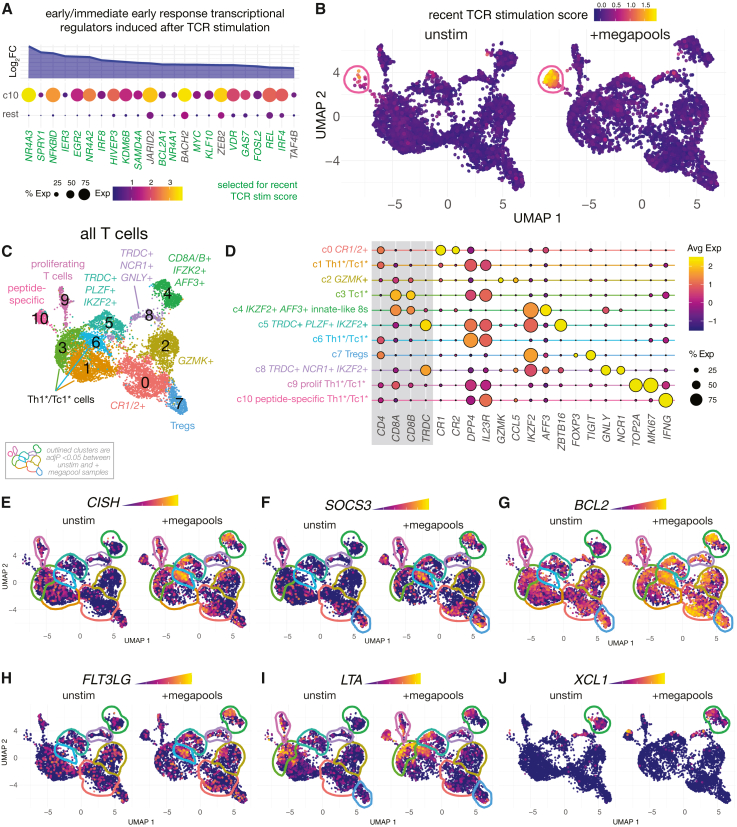
Peptide recognition by Mtb-specific granuloma T cells drives effector responses in bystander and innate-like T cells (A) Expression of selected early and immediate-early response genes that were differentially expressed higher in cluster 10 cells compared to other T cells. Gene names highlighted in green were selected to create a recent TCR stim score. (B) Plot shows this recent TCR stim score in macaque granuloma T cells that cultured without or with Mtb peptide megapools. (C) Granuloma T cells were subclustered and different subsets of T cells were identified by differential gene expression analysis. Well recognized cell populations (e.g., Tregs and Th1∗/Tc1∗ cells) are referred to as common names. Other populations are referred to by their distinguishing features. (D) Dotplot showing selected makers used to describe the clusters of T cells show in C. (E–F) Plots showing gene expression levels of *CISH* (E), *SOCS3* (F), *BCL2*, (G), *FLT3LG* (H), *LTA* (I), and *XCL1* (J) in granuloma T cells cultured either without or with Mtb peptide megapools. Cell clusters are outlined to depict statistically significant comparisons (adjP <0.05) between unstimulated and +megapool cells.

The peptide-specific T cells (cluster 10) also expressed early and immediate-early response genes downstream of TCR signal transduction (Figure 2A). These were mostly transcription factors and chromatin-modifying molecules (e.g., *NR4A1/2/3*, *EGR3*, *NFKBID*, *IER3*, *IFR8*, *KDM6B*, *MYC*, *VDR*, and others). We chose 19 of the most selectively expressed early response genes to create a “recent TCR stimulation score.” As expected, cluster 10 displayed high levels of recent TCR stimulation (Figure 2B). We did not find any other T cell population with high levels of the TCR stimulation score. It is not clear from these data how many granuloma T cells are specific for Mtb peptides not represented in the megapool preparations used here, so we cannot be certain of the precise magnitude of the Mtb peptide-specific T cell response. Nonetheless, for simplicity here we will refer to megapool non-responsive conventional T cells as bystanders.

### Mtb-specific granuloma T cells drive responses in bystander cells

In addition to the megapool-specific T cells (cluster 10), the rest of the T cell population consisted of bystander Th1^∗^/Tc1^∗^ cells (clusters 1, 3, and 6); Tregs (cluster 7); innate-like CD8 T-cells expressing *IKZF2* and *AFF3* (cluster 4); and populations characterized by *GZMK* (cluster 2) or *CR1/CR2* (cluster 0) (Figures 2C and 2D). We also observed two *TRDC^+^IKZF2^+^* populations of cells further distinguished by *ZBTB16* (cluster 5) or *NRC1* and *GNLY* expression (cluster 8). The detection of *TRAC* and *TRDC* mRNAs in the same cells may be due to the nesting of the *TRDC* gene completely within the *TRAC* locus, so clusters 5 and 8 could be γδT cells. However, the precise identities of these clusters are difficult to establish in our dataset.

Upon megapool stimulation, cluster 1 decreased and clusters 10 and 6 increased (Figure S1). Since this was only a 3-h stimulation, the appearance of new clusters after stimulation is due to cells shifting their position on the uniform manifold approximation and projection (UMAP) due to substantial transcriptional changes, rather than proliferation. We do not have information in the TCR sequences which would help us more definitively track movements of T cells from one location on the UMAP to another after stimulation. Therefore, to estimate the sources of the cluster 10 cells that appear after stimulation, we determined which clusters of unstimulated cells were the most frequent nearest neighbors of stimulated cluster 10 in principle component analysis (PCA) space (Figure S1C). This showed that unstimulated clusters 1 and 3 are most similar to stimulated cluster 10. Therefore, the megapool-specific T cells (cluster 10) may be derived from a subpopulation of Th1^∗^/Tc1^∗^ cells in clusters 1 and 3 that shift their position in the UMAP after TCR triggering.

To examine the source of cluster 6, we estimated what clusters of cells in the unstimulated wells most resemble cluster 6 in the stimulated wells in PCA space (Figure S1D). Unstimulated cluster 1 was very similar to stimulated cluster 6, indicating that after stimulation cells from cluster 1 may shift their position in the UMAP creating cluster 6. We next compared the gene expression profile of stimulated (stim) cluster 6 with that of its predicted precursor cells, unstimulated (unstim) cluster 1. We found that after peptide stimulation, cluster 6 upregulated several genes associated with responses to cytokines such as *CISH*, *BCL2*, *SOCS3*, and *CD274* (PD-L1) (Figure S1E). Thus, cluster 6 is a population of Th1^∗^/Tc1^∗^ cells that lacks any indication of a TCR-driven response and instead displays a potent cytokine-driven response. This is consistent with a recent study that found a strikingly similar population of T cells that makes bystander responses to cytokines produced after stimulation of SARS-CoV-2 peptide-specific T cells, confounding the identification of antigen-specific T cells using the activation-induced marker flow cytometry assay.^35^ Our data do not formally exclude the possibility that some cluster 6 cells may be Ag-specific T cells that respond differently to peptide recognition. However, we conclude that cluster 6 is most likely a population of Th1^∗^/Tc1^∗^-like cells that do not recognize the megapool but mount strong bystander responses to cytokines or other signals when megapool-specific T cells are stimulated.

In addition to cluster 6, *CISH*, *SOSC3*, and *BCL2*, were induced in multiple populations of megapool-nonresponsive T cells, indicating that cytokines secreted into the culture by the peptide-specific T cells were able to act on many populations of cells in the well (Figures 2E–2G). Interestingly, polymorphisms in *CISH* have been associated with susceptibility to Mtb infection.^36^^,^^37^^,^^38^^,^^39^^,^^40^^,^^41^ *FLT3LG*, *XCL1*, and *LTA* transcripts were also upregulated on several populations of bystander and innate-like CD8 T cells. Collectively, these data show that TCR stimulation of peptide-specific T cells induces expression of several secreted effector molecules in neighboring T cells (Figures 2H–2J).

We next investigated how megapool-specific T cells might influence bystander T cell states (Figure S2). First, we used CellChat to predict which ligands expressed by the megapool-specific cluster (cluster 10, senders) could engage cognate receptors on every other T cell cluster (receivers). Second, for each receiver cluster we identified stim vs. unstim differentially expressed genes (DEGs)—capturing the transcriptional changes induced in bystanders upon stimulation. Given our previous analysis showing that stim cluster 6 is likely derived from unstim cluster 1, we used DEGS from a comparison of stim c6 vs. unstim c1 for this analysis. Third, for every cluster 10 ligand flagged by CellChat we used NicheNet to compute the Pearson correlation between that ligand’s predicted target-gene profile and the receiver’s experimentally measured up-regulated DEG set. To estimate the overall magnitude of the bystander response, we z-normalized the Pearson scores and summed them per receiver cluster. This analysis showed that cluster 6 and cluster 1 Th1^∗^/Tc1^∗^ cells, *TRDC^+^PLZF^+^IKZF2^+^* cells (cluster 5) and *TRDC^+^GNLY^+^NCR1^+^* CD8 T cells (cluster 8) mount the strongest predicted bystander responses to megapool-specific T cell (cluster 10)-derived signals, driven predominantly by *PDGFA* and *ALCAM*-mediated interactions originating from cluster 10 (Figures S2A and S2B). *ALCAM* encodes CD166, the ligand for the costimulatory molecule CD6. *ALCAM* was highly expressed selectively by megapool-specific T cells and *CD6* on the majority of T cells in the granulomas (Figures S2C and S2D). These data do not show that *ALCAM* mediates bystander T cell activation, but they identify it as an interesting candidate.

### Expression of cytotoxic molecules

**Figure 3:**
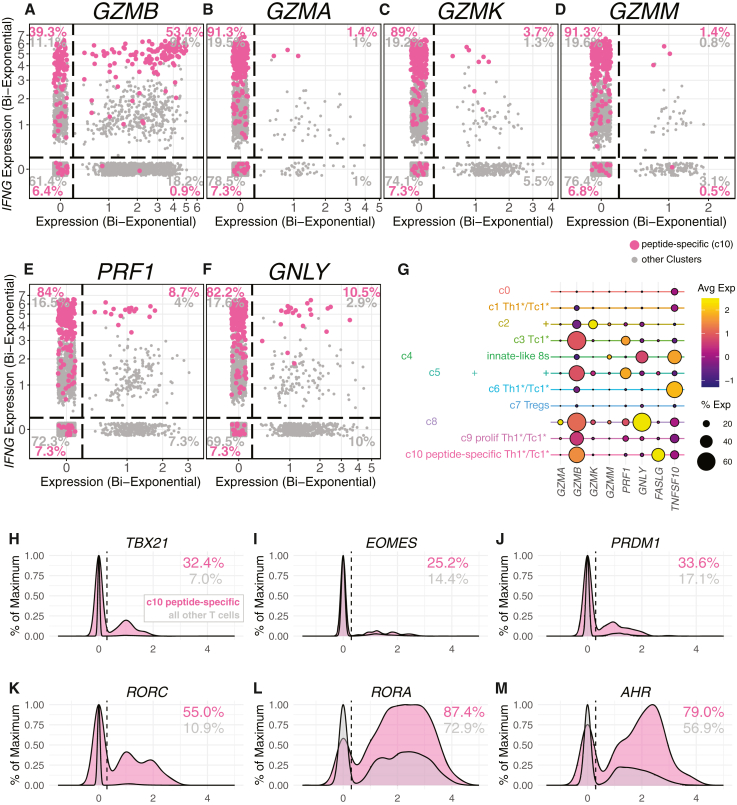
Mtb peptide-specific T cells express *GZMB*, while other populations of bystander and unconventional T cells selectively express *GZMK*, *GZMM*, *PRF1*, and *GNLY* (A–F) Bivariate plots of showing co-expression of *IFNG* with *GZMB* (A), *GZMA* (B), *GZMK* (C), *GZMM* (D), *PRF1* (E), and *GLNY* (F). Cluster 10 cells are highlighted in pink and all other T cells are plotted in gray. To facilitate visualization of gene co-expression and cells with express levels of 0, the axes have been bi-exponentially transformed and jitter added. Numbers represent the percentages of either cluster 10 cells (pink) or all other cells (gray) in each quadrant. (G) Dotplot showing expression levels of selected genes encoding granzymes, cytotoxic, and pro-apoptotic molecules. (H–M) Histogram density plots of the expression levels of the transcripts for the transcription factors *TBX21* (H), *EOMES* (I), *PRDM1* (Blimp) (J), *RORC* (K), *RORA* (L), and *AHR* (aryl hydrocarbon receptor) (M). Cluster 10 cells are shown in pink histograms and all other T cells in gray. Numbers represent the percentage of cluster 10 or other T cells expressing the indicated transcription factor.

We next examined the expression of genes involved in granule-dependent cytotoxicity in peptide-specific and bystander T cells. Approximately 50% of cluster 10 peptide-specific T cells expressed *GZMB* (granzyme B), which was co-expressed with *IFNG* (Figure 3A). However, peptide-specific T cells expressed very little *GZMA*, *GZMK*, *GZMM*, *PRF1*, and *GNLY* (Figure 3B–3F). *GZMK* expression was highest in cluster 2, which was low for other cytotoxicity markers (cluster 2) (Figure 3G). *TRDC^+^PLZF^+^IKZF2^+^* cluster 5 cells expressed many cytotoxic molecules but were enriched for *GZMB* and *PRF1* (Figure 3G). *NRC1*^+^ cluster 8 cells were also notable for the expression of many cytotoxic molecules and high levels of *GZMB* and *GNLY* (Figure 3G). Although peptide-specific T cells did not express a wide range of granule dependent cytotoxic molecules, they did express high levels of *FASLG* and *TNFSF10* (TRAIL), indicating they may be able to induce target cell death with TNF superfamily molecules (Figure 3G). Consistent with their lower expression of cytotoxic molecules, peptide-specific T cells also expressed lower levels of transcription factors required to drive the cytotoxicity program, *TBX21*, *EOMES*, and *PRDM1*, compared to their expression of high levels of transcription factors associated with Th1^∗^ and Th17 polarization, *RORC*, *RORA*, and *AHR* (Figures 3H–3M). These data are also consistent with the observation that human mycobacteria-specific Th1^∗^ cells require *RORC* and not *TBX21* for the production of IFNγ.^15^^,^^17^ Thus, Mtb peptide-specific T cells are polarized more toward a Th1^∗^/Tc1^∗^ vs. killer effector fate, and most T cells with high expression of granule-dependent cytotoxic machinery may be innate-like T cells.

### Comparison of human and rhesus macaque Mtb-specific T cells in granulomas

We next asked if human TB granuloma T cells responded similarly to stimulation with Mtb-peptides. Lung tissue was obtained from a 20-year-old HIV negative male with a right sided hydro-pneumothorax who underwent a pneumonectomy. This individual was diagnosed with pulmonary TB based upon the detection of acid-fast bacilli (AFB) in sputum and a positive GeneXpert test for Mtb DNA. During anti-mycobacterial treatment, the GeneXpert test on his sputum became negative. However, fever and malaise persisted, and his chest radiograph continued to show the collapsed lung. He was transferred to the NIH clinical center eight months after his initial presentation. A chest tube with Heimlich valve was in place, and pleural fluid from the chest tube grew *Achromobacter* species, *Stenotrophomonas maltophilia*, and *Pseudomonas aeruginosa*. GeneXpert testing of the pleural fluid detected Mtb DNA, but no Mtb could be cultured. Given the working diagnosis of entrapped lung with a bronchopleural fistula, the initial surgical plan was pleural decortication to permit lung re-expansion to fill the pleural space. After a month of aggressive management of his malnutrition, he underwent surgery. Intraoperative inspection of the lung revealed extensive inflammation with microabscesses and granulomas involving all three lobes of the right lung. Despite decortication, the lung was solid and unable to expand, so a right pneumonectomy was performed. Pathology of the resected material demonstrated AFB-positive granulomas involving almost the entire right lung parenchyma. The visceral and parietal pleura were fibrous and contained multiple granulomas with AFB. However, all cultures of the resected lung tissue were negative for Mtb growth. The patient required extensive management of subsequent complications which have been reported elsewhere.^42^ The patient has been discharged and remains on treatment with airway clearance measures for post-TB bronchiectasis of the left lung but is otherwise asymptomatic.

**Figure 4:**
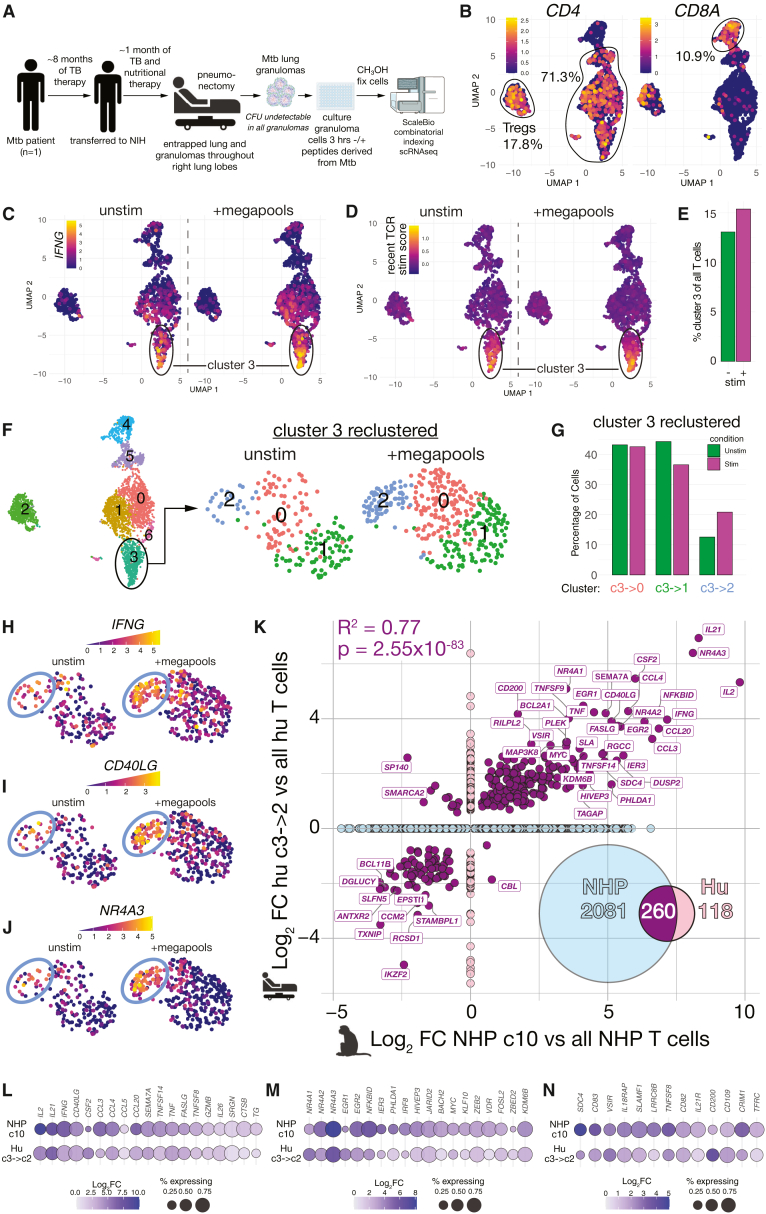
Human and rhesus macaque Mtb-specific T cells in granulomas are functionally similar (A) Individually resected granulomas were obtained from a patient that underwent a right pneumonectomy surgery as a result of tuberculosis. Cells were cultured with or without MTB300 and CD8 Mtb peptide megapools for 3 h. Stimulation was performed alongside the macaque granuloma cells described in the previous figures. Cells were then analyzed by scRNAseq. Cells were sequenced in same run as the rhesus macaque cells. (B) T cells were identified as *TRAC*+ or *CD3E*+ and negative for *CD19*, *MS4A*, *CD68*, *IDO1*, *FLT3*, *FCRL5*, *CD20*, and *CIITA*. Plots show expression levels of transcripts for *CD4*, *CD8A*, and *FOXP3*. Numbers represent the percentage of outlined clusters among all T cells. (C) IFNG expression on human granuloma T cells cultured without or with Mtb peptide megapools. Cluster 3 is noted due to is elevated expression of *IFNG*. (D) Percentage of cluster 3 among T cells that were cultured without or with peptide Mtb megapools. (E) Plots of human granuloma T cells showing the expression levels of the recent TCR stim score developed from peptide stimulation of rhesus macaque T cells as shown in Figures 2A and 2B. (F) Human cluster 3 cells were clustered, yielding 3 new subclusters. (G) Percentage of cluster 3-derived subclusters with and without megapool stimulation. (H–J) Featureplots of *IFNG* (H), *CD40LG* (I), and *NR4A3* (J) expression in cluster 3-derived subclusters. (K) All statistically significant (adjP <0.05) DEGs between human cluster 3->2 vs. all other human T cells were plotted against all statistically significant (adjP <0.05) DEGs between macaque cluster 10 and all other T cells. Venn diagram represents overlap between the two DEG lists. Bivariate plot shows the correlation between genes that were shared between the two lists shown in purple. DEGs only found in the human list are shown in pink and DEGs only found in the macaque list are shown in light blue. (L) Dotplot shows expression of genes encoding selected effector genes that were statistically significant DEGs in both lists. (M) Dotplot shows expression of genes encoding selected early response genes that were statistically significant DEGs in both lists. (N) Dotplot shows expression of genes encoding selected surface molecule genes that were statistically significant DEGs in both lists.

On the day of surgery, single cell suspensions of individually isolated granulomas were obtained from the resected lung material and cryopreserved. No culturable bacteria were detected in the freshly isolated granuloma homogenates by solid culture. In parallel with the macaque samples described above, the human granuloma cells were thawed, rested overnight, FACS-sorted for viable cells, and then cultured with or without Mtb peptide megapools for 3 hours (Figure 4A). Single cell RNAseq analysis was performed alongside the macaque cells, and after filtering, a total of 11,322 human granuloma cells were obtained. Among granuloma T cells, clusters enriched for *CD4* and lacking *CD8A* accounted for 71.3% of the total recovered cells (Figure 4B). A single cluster high for *FOXP3* contained 17.8% of the cells, and a single cluster high for *CD8A* and low for *CD4* represented 10.9% of cells.

Both with and without megapool restimulation, cluster 3 expressed elevated levels of *IFNG* and the recent TCR stim score developed from the macaque T cell stimulation data (Figures 4C and 4D). Unlike what was observed in the macaque cells, this cluster only increased in percentage minimally after stimulation (Figure 4E). We suspected cluster 3 represents T cells already triggered by cognate antigen *in vivo*, but the relatively low increase in *IFNG* and recent TCR stim score after incubation with megapool made it unclear if these cells are Mtb peptide-specific. We further subsetted cluster 3 (Figures 4F and 4G). After stimulation with megapool, the c3->c2 cells increased in frequency, expressed high levels of the cytokines typically used to identify Ag-specific T cells by flow cytometry, *IFNG* and *CD40LG*, as well as the marker of recent TCR stimulation *NR4A3* (Figures 4H–4J). We compared the lists of DEGs in the macaque megapool-specific T cells (cluster 10 vs. all other T cells) with the DEGs in the putative human megapool-specific T cells (c3->2 cells vs. all other T cells) and found a high degree of correlation (R^2^ = 0.77, *p* = 2.55 x10^−83^) (Figure 4K). Given the elevated levels of *IFNG* and TCR stimulation score genes after megapool-stimulation, as well as the similarity with bone-fide peptide-specific T cells from rhesus macaques, we conclude that human cluster 3->2 cells are Mtb peptide-specific T cells.

There was a significant overlap in the potential effector gene expression profile of macaque and human megapool-specific T cells. Cells from both species produced very high levels of transcripts for genes such as *IL2*, *IL21*, *IL26*, *IFNG*, *TNF*, *CCL3*, *CCL4*, *CD40LG*, *CSF2*, and *GZMB*, as well as several molecules less well understood in TB T cell immunology including: *CCL20*, *SEMA7A*, *TNFSF14*, *FASLG*, *CTSB*, *TG*, and *SLAMF1* (Figure 4L). Consistent with the elevated recent TCR stim signature, many early response genes were also shared between the two cells (Figure 4M). There were several other notable transcripts for cell surface molecules shared between the cell types including inhibitory molecules *VISR* (VISTA) and *CD200* and cytokine receptors: *IL21R* and *IL18RAP*. Both species of Ag-specific T cells also expressed *CD109*, *CRIM1*, *and CD82*—encoding proteins which may negatively regulate TGFβ and BMP family signaling (Figure 4N).

## Discussion

Collectively, these data show that peptide-specific *IFNG*^+^ T cells co-express many effector molecules with the potential to influence control of Mtb infection. For example, *IL26*, which has no ortholog in mice, has already been implicated in host resistance to mycobacterial infection and may even be directly antimicrobial.^43^^,^^44^ Likewise, CCL20 is the sole ligand for the Th1^∗^/Th17 cell marker CCR6 and has structural similarity to defensins and the ability to directly kill some microorganisms by binding cardiolipin.^45^^,^^46^ Semaphorin 7A expression by CD4 T cells has been shown to drive macrophage activation through the binding of α1β1 integrin.^47^^,^^48^ SLAMF1 signaling is mediated by homotypic interactions, and SLAMF1 on macrophages has been shown to drive Mtb uptake and endosomal maturation and be important for control of Mtb infection.^49^^,^^50^ SLAMF7, which also operates through homotypic interactions, has been shown to “superactivate” macrophages.^51^ PDGFA is a potent growth factor driving many biological processes and was highly expressed by Mtb peptide-specific T cells. LIGHT (*TNFSF14*), which binds to HVEM and LTβR, may also have a role in control of Mtb infection.^52^ Future studies are needed to directly evaluate the role of these genes in T cell-mediated control of Mtb infection. We should also emphasize that these data show what Mtb peptide-specific T cells can produce under strong stimulation *in vitro*, and the function of Ag-specific T cells recognizing physiological amounts of peptide on the surface of antigen-presenting cells *in vivo* may be more regulated. Understanding the function of macaque and human peptide-specific T cells *in vivo* will require better availability of class I and II tetramers to discriminate between peptide-specific and non-specific bystander T cells without the need for functional assays.

Our data show that while granulomas contain many different types of T cells, most peptide-specific T cells are Th1^∗^/Tc1^∗^-like cells, which have been associated with protection in multiple previous studies.^5^^,^^6^^,^^7^^,^^8^^,^^9^^,^^10^^,^^11^ Our data may not be representative of all the different immune endotypes possible for Mtb-specific immune responses. For example, it has recently been shown that some individuals mount T cell responses characterized by very low expression of IFNγ and skewing toward Th17 and Treg responses.^53^^,^^54^^,^^55^ Many T cells in granulomas may be non-specific conventional bystander and unconventional T cells. Our data show that triggering the TCRs of peptide-specific T cells drives responses in megapool non-responsive granuloma T cells. Several populations upregulated *FLT3LG*, a potent driver of dendritic cells. Innate-like T cells (characterized here by the expression of high levels of *IKZF2*/Helios and sometimes *TRDC*) were induced to express *XCL1*, the ligand for XCR1, which is exclusively expressed by cDC1s. *LTA* was also upregulated on multiple T cell populations after megapool stimulation. These results suggest that conventional peptide-specific T cells may broaden their function and extend their reach by driving effector functions in their abundant bystander and unconventional T cell neighbors.

Granuloma T cells express many different granzymes and cytotoxic molecules. We found a population of unconventional *TRDC^+^*IKZF2^high^*NCR1*^high^ that express very high levels of granulysin and a population of *TRDC^+^IZKF2*^high^*ZTBT16*^high^ cells that expressed the highest levels of perforin. We also found a population of T cells that selectively expressed *GZMK*. Interestingly, several reports have observed an abundance of granzyme K^+^ T cells enriched at peripheral sites of inflammation, but their role in Mtb infection is not well understood.^56^^,^^57^^,^^58^^,^^59^ Peptide-specific T cells, on the other hand, primarily expressed *GZMB*. Only ∼9% of peptide-specific T cells had transcripts for *PRF1* and only 10% for *GNLY*. Peptide-specific T cells did express high levels of transcripts for *FASLG*, which may contribute to target cell apoptosis. It is also possible, however, that these molecules may have functions independent of cytotoxic activity. For example, the engagement of FASLG with FAS can trigger either cell death or inflammatory responses in the FAS positive cell depending on the co-expression of other proteins.^60^ Moreover, granzyme K was recently shown to be a major inducer of complement activation.^61^ Collectively, these data indicate that peptide-specific T cells (primarily *CD8A*^+^ T cells) may mediate some degree of cytotoxic activity, but γδ T cells in the granuloma may be more potent killers.

In the human granulomas the abundance of unstimulated T cells with a high level of *IFNG* expression and recent TCR stimulation score indicates that a substantial fraction of cells may have been actively recognizing antigen *in situ* at the time of resection. Multiple tests found the presence of bacterial DNA and AFB staining organisms, but no bacteria could be cultured, even directly from freshly isolated granulomas. Nonetheless, the lung damage in this patient resulted in a right pneumonectomy. We suggest that, although viable bacteria were not detected in this individual, high levels of Mtb antigens persisted driving damaging effector responses by peptide-specific T cells. Thus, these data are consistent with the hypothesis that chronic stimulation of T cells by antigen from dead, disabled and dormant Mtb bacteria may contribute to the pathology of post-TB lung disease.

For several decades T cell function in TB has been essentially equated with their ability to produce a single molecule, IFNγ. These data show that Mtb peptide-specific Th1^∗^/Tc1^∗^ cells produce a diverse array of molecules that may directly control many physiological processes in granulomas as well as indirectly by driving responses in bystander and unconventional T cells. Additional studies are needed to quantify the role of each of these pathways in host resistance in Mtb infection, as well as to determine peptide-specific granuloma T cell correlates of susceptibility or resistance.

### Limitations of the study

There are several caveats to this study. We only used three macaques and one patient; so, we may have not captured the full diversity of Mtb peptide-specific T cell functional responses. These cells used here were cryopreserved, so we cannot rule out the possibility that we missed some T cell functions mediated by cells that did not survive the preservation process. Also, we only stimulated for three hours, so we may have missed some genes that are more slowly transcribed (e.g., *IL4* and *IL10*). The animals used here were from the control group of a larger drug treatment study and were treated with an isotype control antibody. Lastly, the presence of other co-infecting bacteria in the pneumonectomy patient may have had an impact on the function of Mtb peptide-specific T cells.

## Resource availability

### Lead contact

Requests for further information and resources should be directed to, and will be fulfilled by, the lead contact, Daniel L. Barber, (barberd@nih.gov).

### Materials availability

All materials are available upon request, without restriction, from the lead contact.

### Data and code availability

• Single cell RNA sequencing datafiles are deposited in the GEO repository under the accession number GSE308949.
• This paper does not report original code.
• All other data are available upon request from the lead contact.

## Acknowledgments

The authors would like to thank the Tuberculosis Imaging Program of NIAID, Comparative Medicine Branch of NIAID, Division of Veterinary Medicine, our patient and his family, and the excellent multidisciplinary team who provided his care. This work is supported by 10.13039/100000052NIH/10.13039/100000060NIAID/DIR. The contributions of the NIH authors were made as part of their official duties as NIH federal employees, are in compliance with agency policy requirements, and are considered works of the United States Government. However, the findings and conclusions presented in this manuscript are those of the authors and do not necessarily reflect the views of the NIH or the US Department of Health and Human Services. Christine E. Nelson is an iTHRIV Scholar and is supported in part by the 10.13039/100006108National Center for Advancing Translational Sciences of the National Institutes of Health under award numbers UL1TR003015 and KL2TR003016.

## Author contributions

Conceptualization, C.E.N. and D.L.B.; data curation, C.E.N., K.D.K., K.C.O., A.Shamsaddini, J.Lack, and D.L.B.; formal analysis, C.E.N. and D.L.B.; funding acquisition, D.L.B.; investigation, C.E.N., K.D.K., S.S., J.B., J.M.C., M.C., J.Laux, I.U.A., and A.W.; methodology, C.E.N. and D.L.B.; project administration, C.E.N., K.D.K., A.W., K.P.F., S.R.C., L.E.V., and D.L.B.; resources, K.M.B., I.D., S.C.L.A., A.Sette, J.D.E., C.G.F., L.E.V., K.P.F., S.R.C., and D.L.B.; supervision, D.L.B.; visualization, C.E.N. and D.L.B.; writing – original draft, C.E.N. and D.L.B.; writing – review & editing, C.E.N., K.D.K., K.C.O., S.S., J.B., J.M.C., M.C., J.Laux, I.D., K.M.B., I.U.A., C.S.L.A., A.Sette, A.W., A.Shamsaddini, J.Lack, J.D.E., C.G.F., L.E,V., K.P.F., S.R.C., and D.L.B.

## Declaration of interests

Authors declare no competing interests.

## STAR★Methods

### Key resources table

**Table:** 

REAGENT or RESOURCE	SOURCE	IDENTIFIER
**Antibodies**		

Propidium Iodide	BioLegend	Cat#421301
Control antibody rhesus IgG1 (anti-DSP) [DSPR1]	Nonhuman Primate Reagent Resource	RRID:AB_2716330

**Bacterial and virus strains**		

*Mycobacterium tuberculosis I H37Rv*	Barber Lab stock	H37Rv

**Biological samples**		

Rhesus macaque granulomas single cell suspensions	NIH animal protocol LPD 25E	Animal ID #DI4D, DI5F, DI6N
Human granuloma single cell suspensions	NIH IRB protocol	06C0014 and 93I0119

**Chemicals, peptides, and recombinant proteins**		

MHC-I Mtb300 peptide pool	da Silva Antunes et al.^34^	MHC-I MTB300
MHC-II Mtb300 peptide pool	da Silva Antunes et al.^34^	MHC-II MTB300

**Critical commercial assays**		

ScaleBio Sample Fixation Kit	ScaleBiosciences	Cat#2020001
ScaleBio Single cell RNA sequencing Kit v1.1	ScaleBiosciences	Cat#950884
ScaleBio Single Cell RNA Extended Throughput Kit v1.1	ScaleBiosciences	Cat#936360

**Deposited data**		

Granuloma single cell RNA sequencing data	This paper	GSE308949

**Experimental models: Organisms/strains**		

Rhesus macaques (Indian origin)	NIH	Animal ID DI4D, DI5F, DI6N

**Software and algorithms**		

R/R Studio	N/A	N/A
Seurat	Satijalab.org/seurat	V5.0.0
ScaleBio Seq Suite RNA workflow	github.com/ScaleBio	ScaleRna
CellChat	github.com/sqjin/CellChat	V2

### Experimental model and study participant details

#### Animal models

##### Rhesus macaques

Three healthy male Indian-origin rhesus macaques aged ∼3 years (animal identifications DI4D, DI5F and DI6N) from the National Institute of Allergy and Infectious Diseases breeding colony on Morgan Island, South Carolina were used in this experiment. These monkeys also served as the control group for another experiment that will be reported in a separate manuscript. As part of that study, these animals were treated with isotype control rhesus macaque IgG (Nonhuman Primate Reagent Resource RRID:AB_2716330) at 10 mg/kg on weeks 0, 2, 4, 6, 8, 9, 10, 11, and 12 via intravenous infusion and intratracheal instillation. For all procedures, animals were anesthetized with ketamine and dexmedetomidine and closely monitored for heart rate, respiratory rate, body temperature, and oxygen saturation. Animals were singly housed in nonhuman primate biocontainment racks in a fully Association for Assessment and Accreditation of Laboratory Animal Care (AAALAC) International–accredited Animal Biosafety Level 3 (ABSL3) vivarium at the NIH. Animals were maintained in accordance with the Animal Welfare Act, the Guide for the Care and Use of Laboratory Animals, and all applicable regulations, standards, and policies. Procedures were conducted as outlined in the NIAID Division of Intramural Research Animal Care and Use Committee-approved Animal Study Proposal LPD-25E. Euthanasia of the research animals was performed in accordance with the American Veterinary Medical Association Guidelines. Influence of sex on results could not be evaluated because all animals were male.

#### Human participants

The single patient was a 20-year-old male from The Gambia. A summary of the case report was provided in the results. A full report describing the presentation and post-pneumonectomy management of the case has been reported.^42^ All patient samples were collected under the Institutional Review Board-approved investigational protocols NIH IRB: 06C0014 and 93I0119 and written consent was obtained for the study. Because this study involved a single patient, the influence of sex on results could not be evaluated.

#### Bacterial cultures and strains

*Mycobacterium tuberculosis* H37Rv stocks were generated in house using 7H9 media (MilliporeSigma Cat#M0178) supplemented with OADC (MilliporeSigma Cat#M0678) and Tween80. A 50% glycerol stock was maintained for experimental use. Bacterial stock was diluted in PBS for inoculation and dose verified by plating of 7H11 agar (MilliporeSigma Cat#M0428) plates containing Penicillin G (MilliporeSigma Cat#PENNA).

### Method details

#### NHP Mtb infections

Rhesus macaques were anesthetized with ketamine and dexmedetomidine and 2 mL of physiological saline containing ∼25 CFU of Mtb H37Rv was bronchoscopically instilled into the lower right lobe of the lungs. Aliquots of the inoculum were plated on 7H11 agar plates (MilliporeSigma Cat# M0178) supplemented with OADC (MilliporeSigma Cat#M0678), Tween80 (MilliporeSigma Cat#P1754), and PenG (MilliporeSigma Cat#PENNA) to confirm infection dose.

#### Granuloma processing and cryopreservation

Rhesus macaque necropsies were performed between weeks 13 and 14 post-infection. The lungs of the rhesus macaques were resected and five granulomas from each animal were individually dissected from infected lung tissue. A large section of the patient’s resected lung tissue was also manually dissected for granulomas. Granulomas were homogenized into single cell suspension by manual dissociation and passing through a 100-micron cell strainer (Falcon cat #352360). Single cell suspensions from each individual were washed, counted, and pooled and 2-4.5x10^6^ live cells total. Cell pellets were cryopreserved in 1–2 aliquots of 1 mL of CryoStor CS10 (STEMCELL TECHNOLOGIES).

#### *Ex vivo* peptide megapool stimulation

Cryopreserved granuloma samples were thawed in a 37°C water bath just until liquid phase change was observed. Warmed X-VIVO15 media +10% FBS was added dropwise to the semi-thawed samples with a 1 mL pipet and mixed up and down, slowly, until fully thawed. The thawed sample was added to 9 mL prewarmed media and centrifuged at 400g for 10 min. The supernatant was removed, and the cell pellet resuspended gently in 10 mL of prewarmed media then centrifuged again at 400g for 10 min. Each sample was resuspended in a final volume of 500uL X-VIVO15 media +10% FBS. Samples were rested at 37°C + 5% CO_2_ for 16 h. Rested samples were counted and stained with 5uL propidium iodide (BioLegend cat #421301) for 5 min prior to FACS sorting. PI negative cells (live) were captured, counted, and split into two wells, stimulated and unstimulated, containing 200uL X-VIVO15 media +10% FBS. Stimulated samples also received MHC-I and MHC-II MTB300 Mtb peptide megapools which were added at 1 μg/mL and 2 μg/mL, respectively. Samples were incubated at 37°C + 5% CO_2_ for 3 h. After incubation, samples were centrifuged for 500g for 5 min. The supernatant was removed, and the cell pellet was resuspended in 500 μL ice-cold 1X PBS with wide bore 1 mL pipet tips. Stimulated cells were then prepared for fixation.

#### ScaleBiosciences sample fixation protocol and cryopreservation

Granuloma samples were processed with the ScaleBio Low Input Fixation protocol for single cell RNA sequencing with the ScaleBio Sample Fixation Kit (Scale Biosciences #2020001) with additional fixation time required for BSL3 procedures, as follows. Samples were centrifuged at 500g for 5 min and resuspended in 50 μL 1X PBS in DNA Lo-bind tubes. Scale Bioscience complete fixation reagent was prepared by adding 50 μL of Reconstituted Fixation Reagent +20 μL DEPC to 2 mL of ice-cold methanol. 200 μL of the complete fixation reagent was added dropwise to the 50 μL of sample with gentle tube agitation between drops. Samples were incubated on ice for 1 h. After incubation 500 μL of the Wash Buffer (kit Module A) was added slowly with gentle agitation. Cells were centrifuged for 500g for 5 min. Supernatant was removed and washed again with 500 μL of Wash Buffer, centrifuging at 500g for 5 min. Supernatant was carefully removed and fixed samples were resuspended in 50 μL of wash buffer and frozen.

#### Single cell RNA sequencing and analysis

Fixed frozen samples were thawed on ice, counted, resuspended in Wash Buffer at 2000 cells per μL and processed with the ScaleBio Single cell RNA sequencing Kit v1.1 (Cat #950884) and laboratory protocol RevA NOV2023 from ScaleBiosciences. Approximately 10,000 cells were added per well to the RT-barcode plate on ice. Similar samples were added across rows. Human NS = row A. Di6N NS = row B. Di5F NS = row C1-10, Di4D NS = row D, Human stim = row E and row C11-12, Di6N stim = row F, Di5F stim = row G, Di4D stim = row D. NS = not stimulated.

Two final distribution plates were prepared using ScaleBio Single Cell RNA Extended Throughput Kit v1.1 (Catalog# 936360). 1,600 cells were added per well for approximately 300,000 cells total. Tagmentation was performed with i7-1 (PN 936001) or i7-3 (PN 936003) adapter primers for each plate. Each plate was processed separately for PCR purification and SPRIselect cleanup. The sample was combined and sequenced using NextSeq X Plus with 100 cycles, 150bp paired end, for 10 billion reads.

Fastq files were pre-processed and filtered using ScaleBio Seq Suite: RNA, a cloud-based pipeline on the basepair cloud using nextflow version 23.10.0 and Mmul_10 for rhesus macaque or GRCh38 for human as the reference genome. The Seurat standard preprocessing and clustering workflow was performed for dimensionality reduction and clustering. Briefly, raw counts were normalized, and the top 2,000 highly variable genes were selected using Seurat’s FindVariableFeatures with vst. RunPCA was performed on the variable genes and an SNNgraph was created with FindNeighbors with 20 principal components as determined by an Elbowplot. Clusters were generated with Findclusters and RunUMAP was used to produce the visualization. Fastq files were pre-processed and filtered using ScaleBio Seq Suite RNA workflow: a cloud-based pipeline (github.com/ScaleBio). Filtered barcode, features, and matrix files were used to create rds objects then merged with unique identifiers. Data were analyzed and statistical testing was performed using Seurat V5.0.0 for both macaque and human samples.

### Quantification and statistical analysis

Statistical significance was calculated in R/RStudio using the Seurat pipeline. Data were considered significant after multiple hypothesis testing and adjusted *p* value <0.05.

#### Additional resources

The single patient was not part of a clinical trial. The individual was seen at NIH and sample collected under IRB protocols: 06C0014 and 93I0119.

## Supplemental information

Document S1. Figures S1 and S2

Data S1. *Rhesus macaque* granuloma T cell differentially expressed genes

Data S2. *Rhesus macaque* granuloma T cell differentially expressed genes stim vs. unstim

Data S3. Human granuloma T cell differentially expressed genes

Data S4. *Rhesus macaque* granuloma T cells stim cluster 10 vs. all other clusters

Data S5. Human granuloma T cells stim cluster3->2 vs. all T cells
