# Supplementary material for: Mycobacterium tuberculosis peptide-specific T cells in pulmonary granulomas display broad effector functions
Source: iScience. 2025 Nov 12;28(12):114034. doi: 10.1016/j.isci.2025.114034 (PMC12721174; doi:10.1016/j.isci.2025.114034)
Supplement: Document S1. Figures S1 and S2 [file mmc1.pdf]

iScience, Volume *28*

Supplemental information

Mycobacterium tuberculosis peptide-specific T cells in pulmonary granulomas display broad effector functions

Christine E. Nelson, Keith D. Kauffman, Kevin C. Osum, Shunsuke Sakai, Jay Buchanan, Jean M. Chanchu, Melanie Cohen, Julie Laux, Iyadh Dougi, Katherine M. Barrows, Ifeanyichukwu U. Anidi, Cecilia S. Lindestam Arlehamn, Alessandro Sette, April Walker, Amirhossein Shamsaddini, Justin Lack, Joel D. Ernst, Carl G. Feng, Laura E. Via, Kevin P. Fennelly, Shamus R. Carr, and Daniel L. Barber

Figure S1.

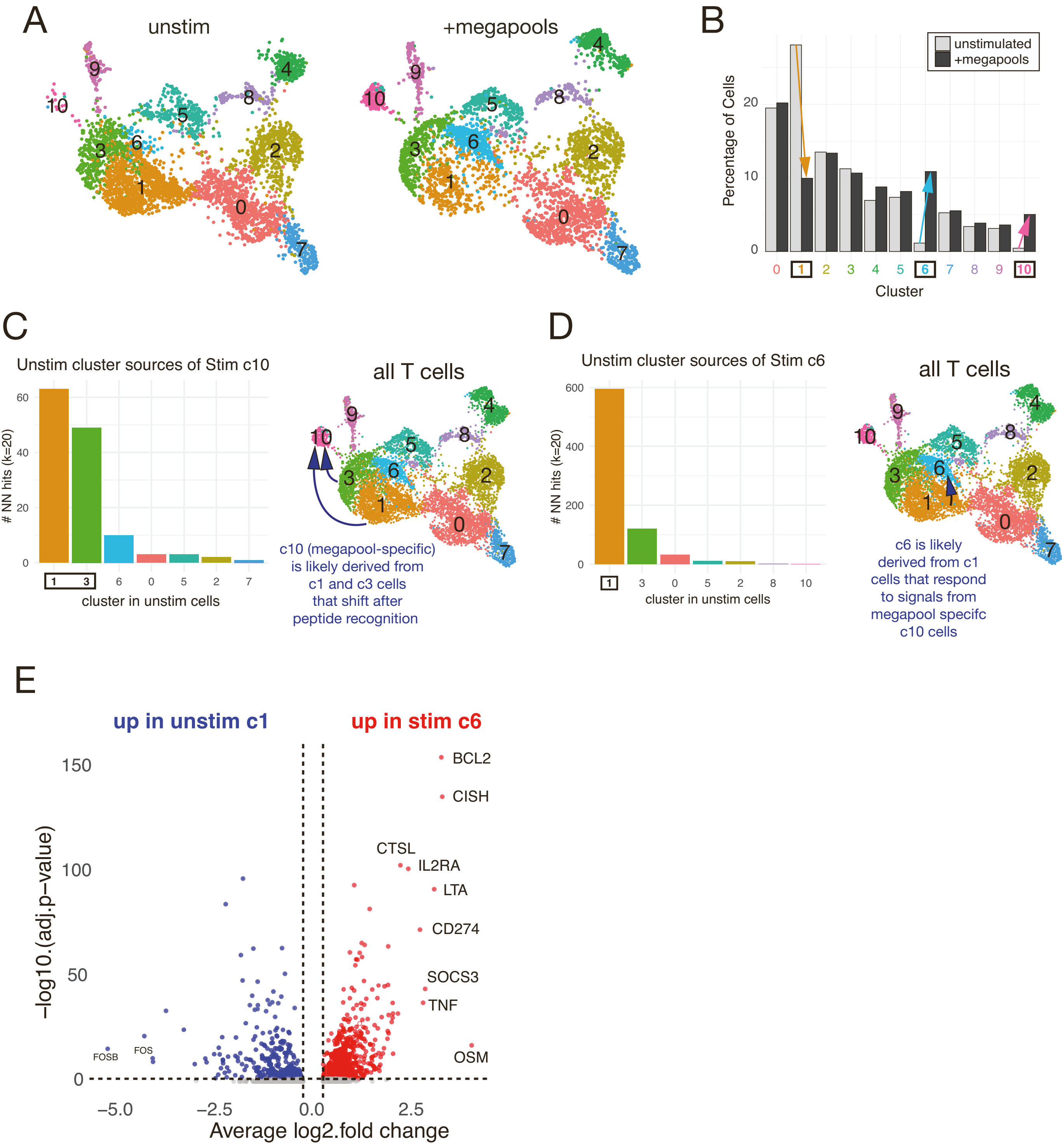
A
unstim
+megapools
B
unstimulated
+megapools
Percentage of Cells
Cluster
C
Unstim cluster sources of Stim c10
NN hits (k=20)
cluster in unstim cells
all T cells
c10 (megapool-specific) is likely derived from c1 and c3 cells that shift after peptide recognition
D
Unstim cluster sources of Stim c6
NN hits (k=20)
cluster in unstim cells
all T cells
c6 is likely derived from c1 cells that respond to signals from megapool specifc c10 cells
E
up in unstim c1
up in stim c6
BCL2
CISH
CTSL
IL2RA
LTA
CD274
SOCS3
TNF
OSM
FOSB
FOS
−log10.(adj.p−value)
Average log2.fold change

Figure S1. Shifts in T cell cluster proportions after peptide megapool stimulation of rhesus macaque granuloma cells. A. UMAP of T cells after a 3 hour culture with or without Mtb peptide megapools. B. Percentage of T cells in each cluster. C - D. A k-NN backtracking approach was used to determine what cluster of cells in the unstimulated wells most likely were the source of the cluster 10 and cluster 6 populations that appeared after stimulation. For stimulated cluster 10 (Panel C) and cluster 6 (Panel D), we determined the k=20 nearest neighbors in PCA space and plot the distribution of those neighbors across the unstimulated clusters. E. DEG analysis of unstimulated cluster 1 and simulated cluster 6, to compare stim cluster 6 with its likely precursor cluster.

Figure S2.

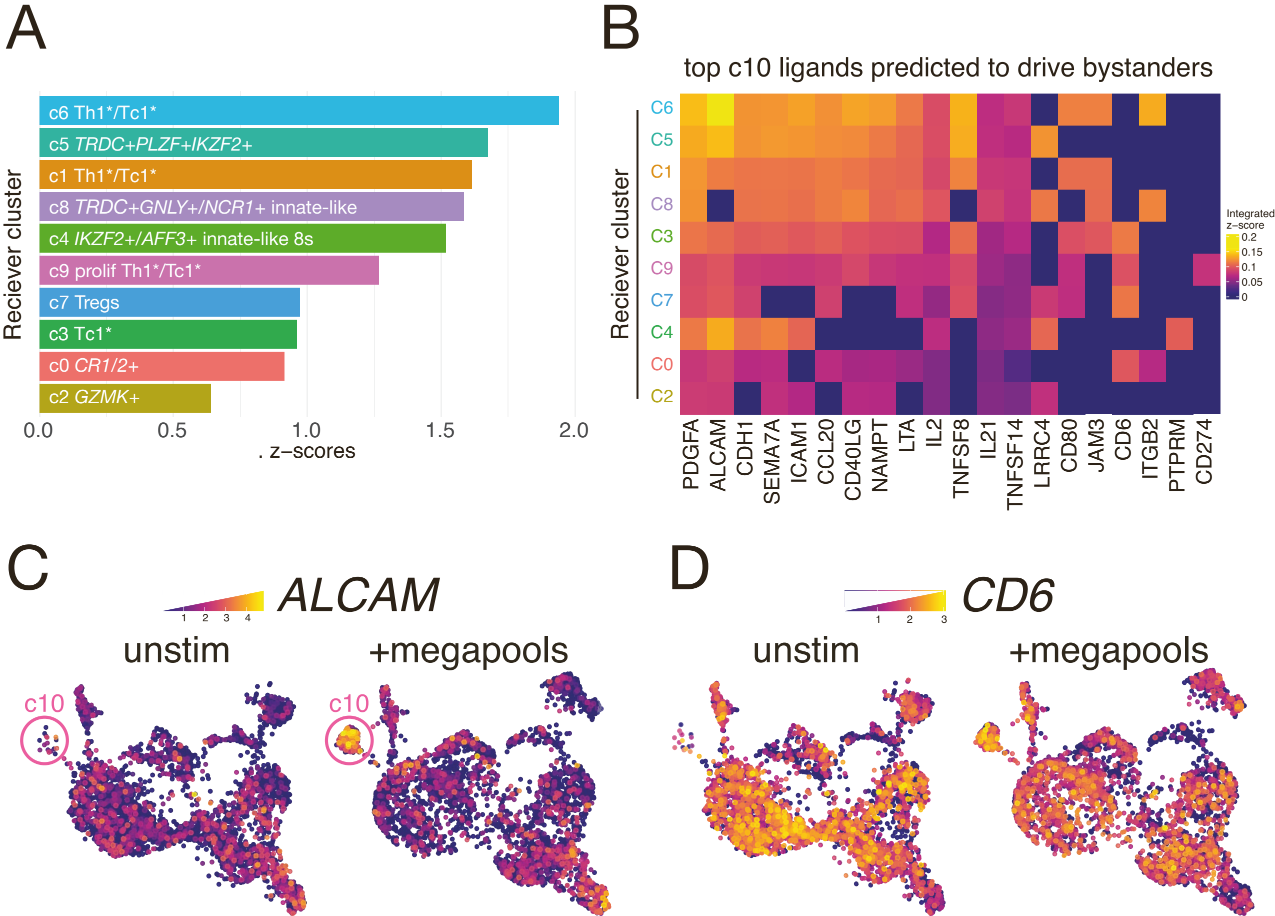
A
B
top c10 ligands predicted to drive bystanders
Reciever cluster
c6 Th1*/Tc1*
c5 TRDC+PLZF+IKZF2+
c1 Th1*/Tc1*
c8 TRDC+GNLY+/NCR1+ innate-like
c4 IKZF2+/AFF3+ innate-like 8s
c9 prolif Th1*/Tc1*
c7 Tregs
c3 Tc1*
c0 CR1/2+
c2 GZMK+
0.0
0.5
1.0
1.5
2.0
. z−scores
Reciever cluster
C6
C5
C1
C8
C3
C9
C7
C4
C0
C2
PDGFA
ALCAM
CDH1
SEMA7A
ICAM1
CCL20
CD40LG
NAMPT
LTA
IL2
TNFSF8
IL21
TNFSF14
LRRC4
CD80
JAM3
CD6
ITGB2
PTPRM
CD274
Integrated z−score
0.2
0.15
0.1
0.05
0
C
ALCAM
1
2
3
4
unstim
+megapools
c10
c10
D
CD6
1
2
3
unstim
+megapools

Figure S2. Predicted pathways mediating peptide-specific T cell driven responses in bystander cells in rhesus macaque cells. To dissect how megapool-specific T cells influence bystander T-cell states, we carried out an integrated CellChat / DEG / NicheNet analysis. First, CellChat predicted which ligands expressed by the megapool-specific sender cluster (c10) could engage cognate receptors on every other T-cell cluster. Second, for each receiver cluster we identified stim vs unstim differentially expressed genes to identify the transcriptional changes induced in bystanders upon stimulation. For c6 DEGS vs unstim c1, its likely precursor population were used. Third, for every c10 ligand flagged by CellChat we used NicheNet to compute the Pearson correlation between that ligand's predicted target-gene profile and the receiver's up-regulated DEG set. A. To estimate the overall magnitude of the bystander response, we z-normalized the Pearson scores and summed them per receiver cluster. B Top 10 c10 ligands predicted to drive bystander T cell responses. C. Feature plot of *ALCAM* expression on T cells. D. Feature plot of *CD6* expression on T cells.
